# Unraveling the Central Role of Sulfur-Oxidizing *Acidiphilium multivorum* LMS in Industrial Bioprocessing of Gold-Bearing Sulfide Concentrates

**DOI:** 10.3390/microorganisms9050984

**Published:** 2021-05-01

**Authors:** Anna Panyushkina, Aleksandr Bulaev, Aleksandr V. Belyi

**Affiliations:** 1 Winogradsky Institute of Microbiology, Research Centre of Biotechnology of the Russian Academy of Sciences, Leninsky Ave., 33, bld. 2, 119071 Moscow, Russia; bulaev.inmi@yandex.ru; 2 JSC “Polyus Krasnoyarsk”, Research Center, Poligonnaya Str., 15, 660118 Krasnoyarsk, Russia; belyiav@polyus.com

**Keywords:** *Acidiphilium* *multivorum*, biooxidation, gold-bearing sulfide concentrates, sulfur metabolism, arsenic resistance, acidophilic microbial communities

## Abstract

*Acidiphilium multivorum* LMS is an acidophile isolated from industrial bioreactors during the processing of the gold-bearing pyrite-arsenopyrite concentrate at 38–42 °C. Most strains of this species are obligate organoheterotrophs that do not use ferrous iron or reduced sulfur compounds as energy sources. However, the LMS strain was identified as one of the predominant sulfur oxidizers in acidophilic microbial consortia. In addition to efficient growth under strictly heterotrophic conditions, the LMS strain proved to be an active sulfur oxidizer both in the presence or absence of organic compounds. Interestingly, *Ac. multivorum* LMS was able to succeed more common sulfur oxidizers in microbial populations, which indicated a previously underestimated role of this bacterium in industrial bioleaching operations. In this study, the first draft genome of the sulfur-oxidizing *Ac. multivorum* was sequenced and annotated. Based on the functional genome characterization, sulfur metabolism pathways were reconstructed. The LMS strain possessed a complicated multi-enzyme system to oxidize elemental sulfur, thiosulfate, sulfide, and sulfite to sulfate as the final product. Altogether, the phenotypic description and genome analysis unraveled a crucial role of *Ac. multivorum* in some biomining processes and revealed unique strain-specific characteristics, including the *ars* genes conferring arsenic resistance, which are similar to those of phylogenetically distinct microorganisms.

## 1. Introduction

Gram-negative bacteria of the genus *Acidiphilium* are members of acidophilic microbial communities in ore deposits, commercial bioleaching operation sites, and acid mine drainage (AMD) [1,2,3,4]. The roles of phylogenetically close species *Ac. cryptum* and *Ac. multivorum* in acidophilic communities are considered similar to those of other acidophilic organoheterotrophs. The latter can utilize metabolites secreted by acidophilic auto-, hetero-, and mixotrophic microorganisms, as well as organic components of lysed cells, as carbon and energy sources [4]. One of the *Acidiphilium* species, *Ac. acidophilum*, has been shown to grow autotrophically with reduced inorganic sulfur compounds (RISCs) [5]. *Acidiphilium cryptum* strain DX1-1 isolated from AMD of the DeXing mine (Jiangxi Province, China) is able to grow with organic compounds and inorganic sulfur. However, when sulfur was the sole energy substrate, the growth and sulfur oxidation by the strain DX1-1 were negligible and improved when glucose was added to the culture medium [6]. Among strains assigned to different *Acidiphilium* species, the ferric iron reduction has been demonstrated in oxygen-containing and anoxic acidic environments [7]. Members of the genus *Acidiphilium* can also withstand high concentrations of toxic metals, such as copper, cadmium, nickel, and zinc [8]. These bacteria were recently characterized by a complex lifestyle granted by horizontal gene transfer. Photosynthetic and heavy metal resistance systems, as well as additional pathways for carbon metabolism, are associated with gene acquisitions that expand the genetic diversity of the genus [9]. Results obtained using genomic approaches have shed light on metabolic pathways and resistance determinants of *Acidiphilium* spp. strains. The latter included an environmental isolate *Acidiphilium* sp. PM from Rio Tinto [10,11], *Acidiphilium* sp. JA12-A1 from an iron-oxidizing mixed culture from a pilot plant for bioremediation of acid mine drainage [12], and an iron-reducing strain *Acidiphilium* sp. C61 isolated from iron-rich pelagic aggregates (“iron snow”) formed at the redoxcline of stratified iron-rich lignite mine Lake 77 (Lusatian mining area, east-central Germany) [13]. Complete sequences of the chromosome and eight plasmids of the strains *Ac. cryptum* JF-5 (BioProject PRJNA15753) and *Ac. multivorum* AIU301 (BioProjects PRJNA224116 and PRJDB503) have been deposited in the GenBank databases and compared [14]. Twelve more genomes of *Acidiphilium* spp. can be also found in the GenBank databases. Three *Acidiphilium* genomes (AccI, AccII, and ZJSH63) were recently sequenced, resulting in the complete nucleotide sequence of the strain AccI (a single chromosome and seven plasmids) and draft genomes of the strains AccII and ZJSH63, and deposited in the JGI IMG-ER database [9]. However, the genome of the only one reported sulfur-oxidizing strain DX1-1 assigned to the heterotrophic species *Ac. cryptum* has not been sequenced, although some of its features have been determined by the differential expression of several genes, and the upregulation of three genes involved in sulfur metabolism has been revealed [6].

The object of this research was an acidophilic bacterial strain isolated as a predominant sulfur oxidizer from a bioreactor during the processing of the gold-containing sulfidic concentrate at 38 °C [15]. Biotechnologies for tank biooxidation of sulfidic concentrates using sulfur- and iron-oxidizing acidophilic microorganisms were developed in the 1980s. Since then, biomining approaches have been successfully applied worldwide [16]. Gold-bearing sulfidic concentrates are classified as refractory concentrates, and direct gold recovery from them using cyanidation is insignificant. Tank biooxidation of gold-bearing arsenopyrite concentrates, using acidophilic microbial communities, is among the most efficient commercial biohydrometallurgical processes known to date [17]. Most arsenopyrite biooxidation processes operate at ~38–42 °C and are dominated by the communities of sulfur- and iron-oxidizing microorganisms, such as *Acidithiobacillus* (*At.*) spp., *Leptospirillum* (*L.*) spp., *Sulfobacillus* (*Sb.*) spp., *Ferroplasma* (*F.*) spp., and some other microorganisms [15,17,18,19,20,21,22]. The Olympiada biooxidation plant is located in the Krasnoyarsk Krai (Siberia, Russia) and has been operated by Polyus Krasnoyarsk JSC since 2001. It is one of the largest biooxidation factories in the world that processes from 8 to 9 million tons of gold-bearing ore annually using a high-performance BIONORD® technology developed under the conditions of Extreme North (Siberia, Russia) [15,20]. The average productivity of the Olimpiada plant is 1200–1300 tons of the flotation sulfidic concentrate per day, which allowed the recovery of 30 tons of gold in 2017 [20]. The main minerals composing sulfide concentrates are pyrite, arsenopyrite, pyrrhotite, and antimonite [15].

The goal of our research was to characterize the predominant sulfur-oxidizing bacterium that was isolated from the bioreactors of the Olimpiada plant and subsequently assigned to the heterotrophic species *Ac. multivorum*, as well as to determine its role in the microbial communities during the biooxidation of sulfidic concentrates. Within several years, this bacterium has become one of the predominant sulfur-oxidizing organisms, along with another sulfur-oxidizing species *At. thiooxidans*, which is commonly detected in bioreactors at the Olimpiada plant [21], or even succeeded the latter in some bioreactors. According to the previously obtained results of metabarcoding analysis (V3–V4 variable regions of the 16S rRNA gene), the proportion of the *Acidiphilum* bacteria in the microbial community reached 36% [15]. Therefore, we aimed to study the characteristic features of this acidophile, as well as to determine and analyze its genome sequence to provide new insights into its metabolism capacities, resistance mechanisms, and the role in the biooxidation of the refractory gold-bearing ores. The sulfur-oxidizing activity of this bacterium, together with the sulfur metabolism pathways predicted from the genome annotation, allowed us to conclude that the role of some heterotrophic acidophiles could be underestimated in acidophilic microbial consortia. The strain *Ac. multivorum* LMS proved to be an active sulfur oxidizer that possessed a complicated sulfur-oxidizing enzyme system, including the multi-enzyme Sox complex, to oxidize RISCs. Based on the genome analysis, a biochemical model for sulfur oxidation in *Ac. multivorum* LMS was proposed. A comparison to other *Acidiphilium* spp. genomes revealed additional strain-specific metal(loid) resistance determinants in the LMS strain. Probably, these features determined the crucial role of this microorganism in the industrial biooxidation of sulfidic raw materials.

## 2. Materials and Methods

### 2.1. Strain Isolation and Cultivation Conditions

The phylogenetically close sulfur-oxidizing *Acidiphilium* strains (including the LMS strain) were continuously isolated from the pulp samples collected from industrial bioreactors during the monitoring of the microbial population involved in the biooxidation of the gold-bearing pyrrhotite-containing pyrite-arsenopyrite concentrate at the Olimpiada biooxidation plant (38–42 °C; the pulp density of 15–20%; gold recovery of ~97% after biooxidation) in 2016 [15] and 2017–2019 [23]. The pure cultures of the strains designated LMS, PB-B3-4, PB-B1-3, and PB-B2-3 were isolated from the pulp samples collected from the bioreactors by serial tenfold dilutions in the selective liquid media (pH 2.5) containing elemental sulfur S^0^ (1%, *w*/*v*) and yeast extract (0.02%, *w*/*v*) or only S^0^ (autotrophic medium) at 40 °C. The amount of inoculum was 10% (*v*/*v*). The strains were cultured in 250 mL Erlenmeyer flasks (100 mL of the liquid medium) on Unimax-1010 rotor shakers (Heidolph Instruments, Schwabach, Germany; 180× rpm) in Inkubator-1000 thermostats (Heidolph Instruments, Schwabach, Germany). The salt base of the 9K medium was of the following composition (g/L): (NH_4_)_2_SO_4_, 3.0; KCl, 0.1; KH_2_PO_4_, 0.5; MgSO_4_ 7H_2_O, 0.5; Ca(NO_3_)_2_ 4H_2_O, 0.01 [24].

To determine the characteristics of the LMS strain growth and substrate oxidation, it was cultivated in the temperature range of 20–60 °C (pH 2.5) in the presence of S^0^ (1%, *w*/*v*) and yeast extract (0.02%, *w*/*v*). After determining the optimum temperature, the growth of the strain at different pH was tested. The initial ambient pH value of the medium was adjusted to 1.5–6.5 with 10 N H_2_SO_4_ depending on the experimental conditions, and the variants were cultivated at 38 °C. The 9K base salt media supplemented with Na_2_S_2_O_3_ (2%, *w*/*v*; pH 2.5), Na_2_S_4_O_6_ (2%, *w*/*v*; pH 2.5), or FeSO_4_·7H_2_O (20 mM Fe^2+^; pH 1.9) in the presence or absence of yeast extract (0.03%, *w*/*v*) were used to evaluate the ability of the strain to use RISCs and ferrous iron as energy sources. Yeast extract and/or glucose (0.01–0.5%) were used to test the growth of the LMS strain under heterotrophic conditions. Two series of experiments in each variant of the bacterial growth included three parallels (flasks) and three replicates for measured parameters. Statistical processing was performed using Microsoft Excel 2010. The standard deviation (SD) of the arithmetic mean was calculated, and the significance of the results was assessed using the Student’s *t*-test at the significance level *p* ≤ 0.05.

The biomass of the late-exponential cells grown under auto- and mixotrophic conditions was used to determine the phylogenetic position of the strain by subsequent sequencing of the 16S rRNA gene. For genome sequencing, the biomass of late-exponential cells was grown in the 9K medium containing both S^0^ and yeast extract in 2500 mL Erlenmeyer flasks (1500 mL of the inoculated medium).

### 2.2. Taxonomic Research and Phylogenetic Analysis

To carry out phylogenetic identification of the LMS strain pure culture, the microbial biomass was collected by centrifugation (8000× *g*, 20 min, 4 °C). The DNA extraction and purification, PCR amplification of the 16S rRNA gene fragment with standard primers for the bacterial 16S rRNA gene fragment, amplicon purification and subsequent sequencing, as well as phylogenetic identification of the pure microbial cultures, were carried out as previously described [21]. The 16S rRNA gene sequences of *Acidiphilium* sp. strains were deposited in the GenBank databases under accession numbers MW389315–MW389318. The phylogenetic tree was constructed in the MEGA X software package [25] using the sequences of the type strains and some other isolates identified as the closest *Acidiphilium* strains with the BlastN algorithm (https://blast.ncbi.nlm.nih.gov/Blast.cg; accessed on 8 September 2020). The percentage of replicate trees in which the associated taxa clustered together in the bootstrap test (1000 replicates) is shown next to the branches [26]. The evolutionary history was inferred using the Neighbor-Joining method [27]. The evolutionary distances were computed using the Maximum Composite Likelihood method [28] and were in the units of the number of base substitutions per site.

### 2.3. Genome Sequencing and Assembly

Late-exponential cells of the LMS strain were collected by centrifugation (8000× *g*, 20 min, 4 °C) and washed twice with acidified 9K medium without energy sources (pH 2.5) for the subsequent genome sequencing. Total DNA for genome sequencing was isolated using the FastDNA Spin kit (MP Biomedicals, Irvine, CA, USA) according to the manufacturer’s recommendations. DNA was fragmented using the NEBNext dsDNA Fragmentase (New England BioLabs, Inc., Ipswich, MA, USA). Subsequent steps of library preparation were carried out with the KAPA HyperPlus fragment library kit (Roche, Basel, Switzerland) according to the manufacturer’s protocol. The genome of the LMS strain was sequenced on the Illumina NextSeq platform (Illumina, Inc., San Diego, CA, USA) using the 2 × 100-cycles paired-end sequencing kit. Assembly of reads was performed with the SPAdes v. 3.13.0 (Genome Assembler; Center for Algorithmic Biotechnology: St. Petersburg, Russia, 2018) [29] and resulted in 139 contigs of the total length of 3,837,559 bp and an N50 value of 69,349 bp. The final assembly coverage was 500×.

### 2.4. Genome Annotation and Analysis

The draft genome of *Ac. multivorum* LMS was sequenced using the RAST (Rapid Annotation using Subsystem Technology) service v. 2.0 (RASTtk annotation scheme, https://rast.nmpdr.org/; accessed on 5 August 2020) for annotating microbial genomes [30] and the NCBI Prokaryotic Genome Annotation Pipeline (Bethesda, MD, USA, http://www.ncbi.nlm.nih.gov/genome/annotation_prok/; accessed on 8 September 2020). The genome sequence of *Ac. multivorum* LMS was deposited in the NCBI databases under the accession number JACVVW000000000 (BioProject PRJNA662208; BioSample SAMN16078398). Functional characterization of the genome was carried out using the KOALA (KEGG Orthology And Links Annotation) system [31]. Pathway mapping was carried out using tools of the KEGG software package. The annotation was manually improved by comparison with the type strain *Ac. multivorum* AIU301^T^ (https://www.ncbi.nlm.nih.gov/genome/3581; accessed on 8 September 2020), *Ac. cryptum* JF5 (https://www.ncbi.nlm.nih.gov/genome/1383; accessed on 8 September 2020), and *Acidiphilium* spp. genomes (https://www.ncbi.nlm.nih.gov/genome/browse/#!/prokaryotes/13804/; accessed on 8 September 2020), available in the NCBI GenBank databases, as well as three recently published and characterized *Acidiphilium* genomes (strains AccI, AccII, and ZJSH63) that have been deposited in the JGI IMG-ER database (genome IDs 2824045439, 2824049744, and 2828882166, respectively) [9]. Genomes of *Ac. multivorum* LMS and other *Acidiphilium* strains were compared using the progressiveMauve program and the Mauve Contig Mover tool, which are part of the Mauve software package v. 2.4.0 for multiple genome alignments (The Darling lab at the University of Technology: Sydney, Australia, 2014) [32]. The average nucleotide identity (ANI) values between the genomes of *Ac. multivorum* LMS and other *Acidiphilium* strains were calculated with the ANI calculator tool (Kostas lab, Atlanta, GA, USA) using both best hits (one-way ANI) and reciprocal best hits (two-way ANI) between genomic datasets (http://enve-omics.ce.gatech.edu/ani/index; accessed on 16 April 2021). Circular visualization of the draft genome of the LMS strain and other *Acidiphilium* genomes used for comparisons was performed with the BRIG (BLAST (Basic Local Alignment Search Tool) Ring Image Generator v. 0.95) cross-platform application that can display circular comparisons between a large number of genomes, with a focus on handling genome assembly data (https://sourceforge.net/projects/brig/; accessed on 16 April 2021), and the NCBI BLAST+ database [33]. To identify the strain-specific genes and proteins (novel regions in the genome), we performed a comparison using the Panseq online tool (https://lfz.corefacility.ca/panseq; accessed on 4 December 2020) for analysis of core genome, pangenome, and accessory genomic regions [34]. The amino acid and nucleotide sequences of interest were compared using the NCBI BLAST (Bethesda, MD, USA, https://blast.ncbi.nlm.nih.gov/Blast.cgi; accessed on 16 April 2021).

### 2.5. Analytical Techniques

The pH value and concentrations of Fe^3+^ and Fe^2+^ were measured as previously described [18,21]. The quantitative assessment of the LMS strain was carried out by direct counts and by the method of serial terminal tenfold dilutions. Phase-contrast microscopy was performed with an Olympus CX41 microscope (Olympus Corporation, Tokyo, Japan) equipped with an Olympus C5060-ADU camera (Olympus Corporation, Tokyo, Japan); the ImageScope Lite software package (ImageScope Color Application, Moscow, Russia) was used. Microscopy was also used to determine morphological alterations within the cell population of the strain.

## 3. Results and Discussion

### 3.1. Isolation and Identification of the LMS Strain

*Acidiphilium* spp. strains were first isolated by us among the predominant sulfur oxidizers from a microbial community involved in the active process of biooxidation in different chains of bioreactors at the Olimpiada plant in 2016. The presence of this species in the bioleaching process has been also confirmed by metabarcoding analysis (V3–V4 variable regions of the 16S rRNA gene) of the microbial community structure during the monitoring of the microbial populations of commercial bioreactors in 2016–2019 [15,23]. In total, more than 200 samples have been collected from the bioreactors and analyzed by the 16S rRNA gene metabarcoding over this period, and the maximum proportion of *Acidiphilium* in the total microbial population reached 36–41% depending on the bioreactor [15,23].

Pure cultures of the predominant sulfur-oxidizing microorganisms were isolated under both autotrophic or mixotrophic conditions of cultivation from several bioreactors by a method of tenfold serial dilutions in selective liquid growth media supplemented with elemental sulfur or yeast extract and S^0^. The growth of the cultures was shown until the 10^−7^–10^−9^ dilution, which indicated that the strain abundance in the original pulp samples collected from the bioreactors reached 10^7^–10^9^ cells/mL. The similarity of the nucleotide sequences of the 16S rRNA gene fragments (1423–1456 bp) of the pure cultures isolated from the auto- and mixotrophic culture media containing S^0^ and inoculated with the pulp material from different reactors reached 99.93–100%. The isolates were designated *Acidiphilium* sp. strains PB-B3-4, PB-B1-3, PB-B2-3, and LMS. Their 16S rRNA gene sequences were deposited in the GenBank databases under accession numbers MW389315–MW389318. The closest phylogenetic relatives of these isolates belonged to the species *Ac. multivorum*. Thus, unexpectedly, *Acidiphilium* strains turned out to be sulfur oxidizers dominating the microbial communities during biooxidation of the pyrite-arsenopyrite concentrate. Although this species is frequently identified in the composition of various acidophilic chemolithotrophic microbial communities in AMD and at mining sites [3,4], it is considered to possess an organoheterotrophic type of metabolism [1,2]. One of the most abundant sulfur-oxidizing strains in the microbial community, the LMS strain (up to 10^9^ cells/mL), was used for subsequent studies.

**Figure 1:**
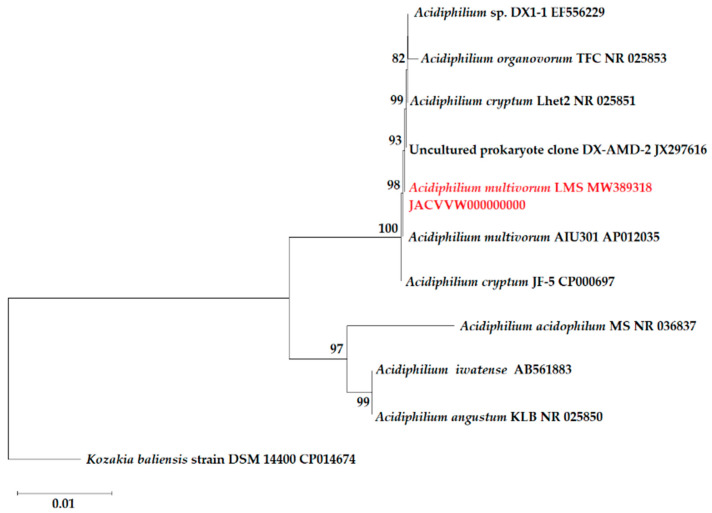
Dendrogram of *Acidiphilium* strains based on the 16S rRNA gene sequences, showing the phylogenetic position of *Ac. multivorum* LMS. The phylogenetic tree was constructed with MEGA X [25]. The evolutionary history was inferred using the Neighbor-Joining method [26]. The percentage of replicate trees in which the associated taxa clustered together in the bootstrap test (1000 replicates) is shown next to the branches [27]. The bootstrap values of ≥80 are shown. The tree is drawn to scale, with branch lengths in the same units as those of the evolutionary distances used to infer the phylogenetic tree. The evolutionary distances were computed using the Maximum Composite Likelihood method [28] and are in the units of the number of base substitutions per site. This analysis involved 11 nucleotide sequences. There were a total of 1511 positions in the final dataset. *Kozakia baliensis* DSM 14400^T^ was used as an outgroup.

Based on the partial and complete 16S rRNA gene sequences of the pure culture of *Acidiphilium* sp. LMS, obtained by Sanger sequencing (GenBank accession number MW389318) and derived from the draft genome sequence of this isolate (GenBank accession number JACVVW000000000), respectively, and the 16S rRNA gene sequences of other *Acidiphilium* spp. available in the GenBank databases, a phylogenetic tree was constructed to show the position of the novel LMS strain among other *Acidiphilium* strains (Figure 1). All analyzed 16S rRNA gene sequences clustered into two distinct subgroups within the genus *Acidiphilium*. The novel strain *Ac. multivorum LMS and the strains* *Ac. multivorum* AIU301 and *Ac. cryptum* JF-5 belonged to the same subgroup (Figure 1). Li et al. have recently carried out a thorough phylogenetic analysis of the *Acidiphilium* spp. strains based on the draft or whole-genome sequences and the 16S rRNA gene sequences [9]. According to this classification, all strains have been grouped into four clades (I–IV) [9], and the LMS strain may be assigned to clade IV, which includes *Ac. multivorum* AIU301, *Ac. cryptum* JF-5, as well as *Acidiphilium* spp. strains AccI, AccII, and ZJSH63.

### 3.2. Phenotypic Features of the Strain Ac. multivorum LMS

Phenotypic characteristics of the novel LMS strain were studied and compared to those of the strains described in the scientific literature. However, the detailed phenotypic characterization is available only for its close phylogenetic relative: the type strain *Ac. multivorum* AIU301^T^ [2]. The main phenotypic features of *Ac. multivorum* LMS are summarized and compared to characteristics of *Ac. multivorum* AIU301^T^ in Table 1.

**Figure 2:**
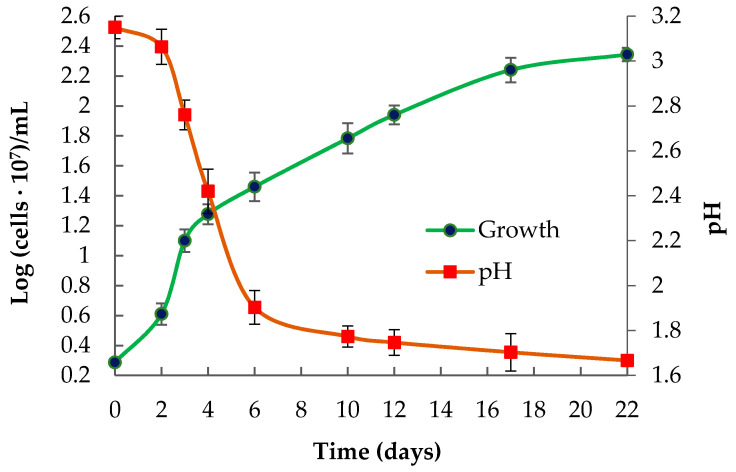
Growth of *Ac. multivorum* LMS and pH decrease under mixotrophic conditions in the medium supplemented with 0.1% yeast extract and 1% S^0^.

According to the results, the LMS strain had wider pH and temperature ranges for growth than the strain AIU301 (Table 1). A higher temperature optimum for growth of the LMS strain (30–40 °C) and a wider pH optimum (pH 2.5–4.0), as well as broader pH and temperature ranges (especially the upper-temperature limit of 47 °C and the lower pH limit of 1.6), may be associated with the competitive advantage of this strain under the conditions of the industrial processing of sulfidic concentrates (38–42 °C, pH 1.8–2.0). Moreover, the type of strain of the species *Ac. multivorum* was not shown to oxidize RISCs, while the novel LMS strain was able to grow in the media containing elemental sulfur or thiosulfate as sole energy sources. Curves for the growth and sulfur oxidation by the LMS strain under optimal mixotrophic conditions of cultivation are shown in Figure 2. Neither other *Ac. multivorum* strains nor *Ac. cryptum* isolates used S^0^ or other RISCs in their metabolism, except for the strain *Ac. cryptum* DX1-1, which showed 99.7% similarity to the LMS strain (according to the 16S rRNA gene sequence comparison). The strain DX1-1 can grow with either organic substances or inorganic sulfur energy substrates [6]. With sulfur as the sole energy source, the strain DX1-1 had a low growth rate and cell density, which, however, increased when a small amount of glucose was added to the medium [6].

**Figure 3:**
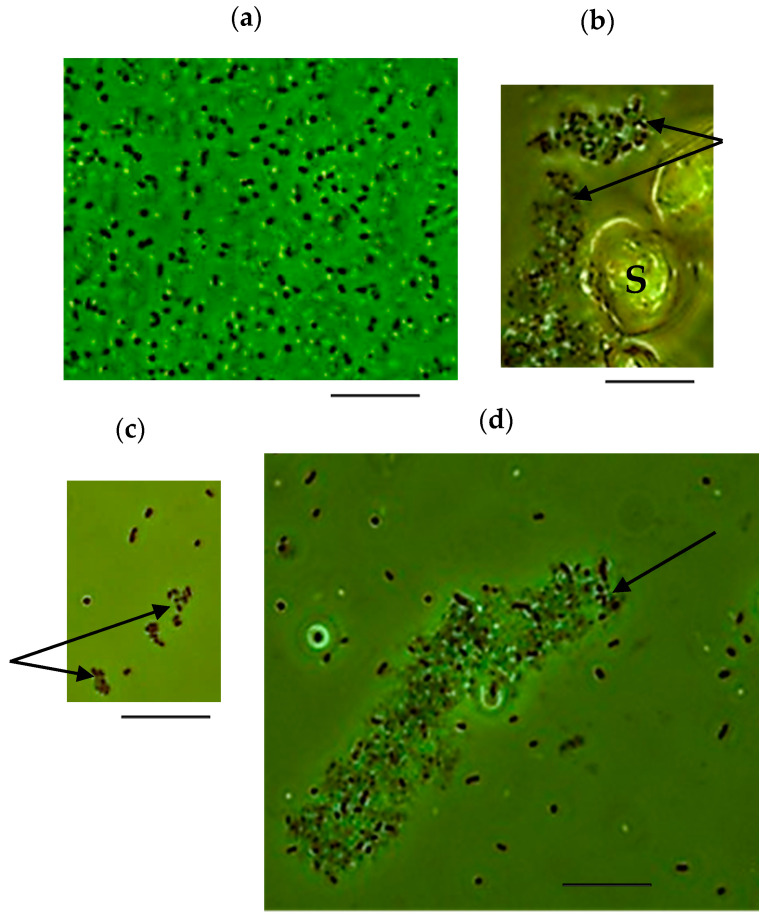
Micrographs of *Ac. multivorum* LMS cells grown under mixotrophic conditions in the medium containing 1% S^0^ and 0.1% yeast extract after isolation from the bioreactor (**a**) and after 5–7 culture transfers in the same medium (**b**–**d**). Phase-contrast microscopy. Arrows indicate cell aggregates. S, sulfur. Scale bar, 10 µm.

Interestingly, the isolated LMS strain was represented by single or dividing cells (Figure 3a); however, after a series of culture transfers, cells tended to aggregate (Figure 3b–d). Under heterotrophic, autotrophic, and mixotrophic conditions, *Ac. multivorum* LMS formed aggregates that consisted of from three to several hundred cells. Such aggregation may be similar to that observed for *Acidiphilium* sp. C61, *Ac. cryptum* JF-5, and *Acidiphilium* SJH [13]. Extracellular polymeric substances (EPS) are considered to be among the major structural components of biofilm matrix and these aggregates, in particular. Genes encoding proteins involved in the synthesis and secretion of exopolysaccharides, such as glycosyltransferases, the polysaccharide biosynthesis/export protein (wza), and the capsular polysaccharide export protein (kps) were identified in *Acidiphilium* strains [13]. In the LMS strain, several glycosyltransferase genes were also found, and Wza homologs were identified: ICJ77_06775 (contig 9) and ICJ77_11025 (contig 20). The presence of different pathways involved in exopolysaccharide precursor production suggests that EPS biosynthesis plays a significant role in *Acidiphilium* sp. C61 aggregate formation [13]. In the case of *Ac. multivorum* LMS, the formation of aggregates may also be associated with sulfur oxidation. Cells can adhere to the sulfur particles forming aggregates on their surface (Figure 3b).

### 3.3. General Description and Specific Features of Ac. multivorum LMS Genome

**Figure 4:**
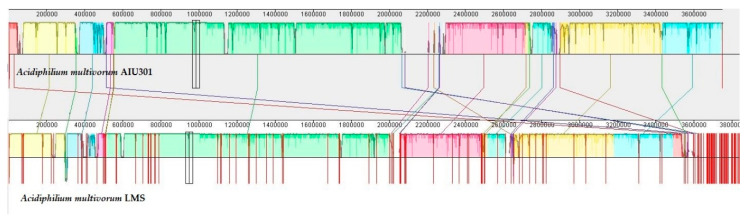
Alignment of reordered contigs of the *Ac. multivorum* LMS draft genome and the related reference genome of the type strain *Ac. multivorum* AIU301^T^, using the ProgressiveMauve software and Mauve Contig Mover tool [32]. The scale shows sequence coordinates. Colored blocks in the first genome are connected by lines to similarly colored blocks in the second genome. These lines indicate homologous regions in the genomes. Areas that are completely white and not aligned contain sequence elements specific to the genomes.

The draft genome sequence of *Ac. multivorum* LMS consisted of 3,837,559 bp with an overall G + C content of 67.3% (Table 2). A total of 3676 genes were predicted. Among them, 3492 coding genes and 131 pseudogenes were identified. Genes with unclear functions were annotated as hypothetical proteins. Functional annotation of proteins encoded by the genes of the LMS draft genome sequence was carried out. According to the functional classification in clusters of orthologous groups (COGs), all identified proteins were grouped into 21 functional categories (Table 3). Figure 4 shows the alignment of the genomes of *Ac. multivorum* LMS and the type strain *Ac. multivorum* AIU301^T^, indicating the close relationship between them and the highly similar metabolic potential, as also previously reported for *Acidiphilium* strains [12].

**Figure 5:**
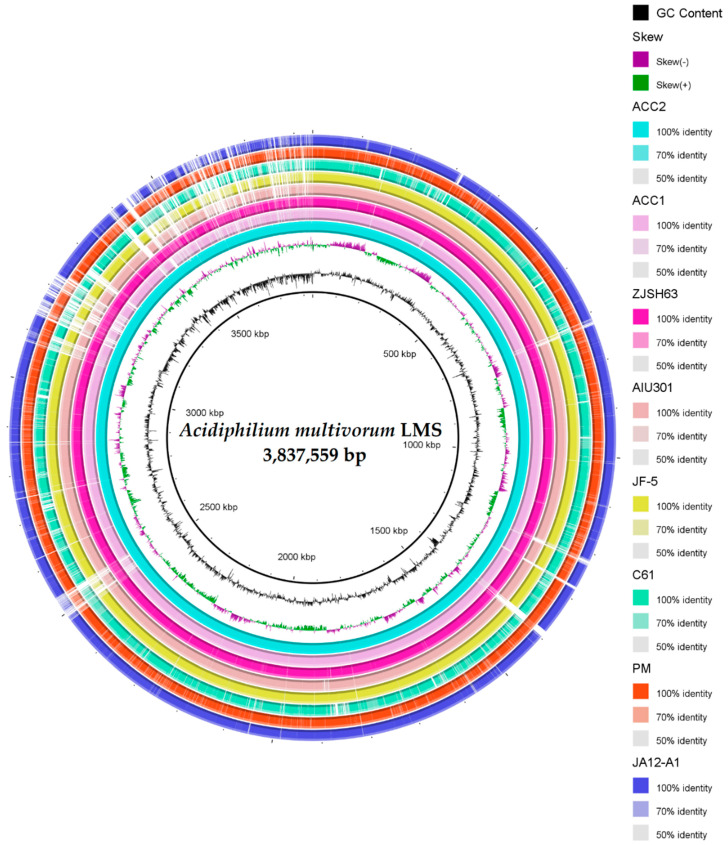
Circular map of the aligned draft genome sequence of *Ac. multivorum* LMS and other *Acidiphilium* genomes, using the BLAST Ring Image Generator (BRIG) software program [33]. The panel on the right shows color codes for different *Acidiphilium* strains and the identity levels (%) with the *Ac. multivorum* LMS genome. ACC2, ACC1, and ZJSH63: *Acidiphilium* spp. AccII, AccI, and ZJSH63, respectively [9]; AIU301 and JF5: *Ac. multivorum* AIU301^T^ and *Ac. cryptum* JF-5, respectively [14]; C61, *Acidiphilium* sp. C61 [13]; PM and JA12-A1: *Acidiphilium* spp. PM [10] and JA12-A1 [12], respectively.

The circular representation of the *Ac. multivorum* LMS draft genome and genomes of other *Acidiphilium* strains used for comparison is shown in Figure 5. Table 4 indicates the average nucleotide identity (ANI) between the genomes of *Ac. multivorum* LMS and other members of the genus *Acidiphilium*, showing the relatedness among *Acidiphilium* strains. Based on the results of this comparison (Figure 5 and Table 4), the genome of the LMS strain showed the highest homology to that of the strain *Acidiphilium* sp. AccII isolated from the acid mine drainage (AMD) water sample obtained in the mining area [9]. At the same time, the strain AccII contained more unique genes with adaptive functions than two other strains that were more similar to each other: AccI (isolated from the same sample as AccII) and strain ZJSH63 from an AMD water sample collected in the heap leaching area for copper ore [9].

*Acidiphilium* genomes that are currently available in databases have been recently compared by Li et al. to provide insights into the metabolism and evolution of the genus *Acidiphilium* [9]. Therefore, in our present study, we compared the genome of the LMS strain to other *Acidiphilium* spp. genomes by analysis of the pangenome, core genome, and accessory genomic regions to focus on the strain-specific features of *Ac. multivorum* LMS. As a result, a total of 32 unique genes and corresponding proteins were found in the genome of this strain (Table S1). The strain-specific genes mainly belonged to the mobile genetic elements (such as transposons, plasmids, and prophages) or encoded the proteins involved in transcription, replication, recombination, and repair (Table S1). A number of unique genes were associated with the metal(loid) resistance mechanisms. Among them, we identified the *arsR*, *arsD, arsA*, and *arsB* genes of the probable *arsRDABC* operon, which composed a 3785-bp contig 98 (Table S1, ICJ77_17765–ICJ77_17785).

In industrial operations, arsenic concentrations may reach >120 mM at pulp densities of 20 wt% [18]. In acidic conditions, arsenic is most commonly present as either arsenate (As^5+^/AsO_4_^3−^) or arsenite (As^3+^/As(OH)_3_). Arsenate enters the cell via the phosphate uptake system and is toxic by acting as a phosphate analog, while the more toxic arsenite enters cells via aquaglyceroporins and some sugar transporters and is toxic by binding sulfhydryl groups [35]. Since arsenic concentrations reach high values during the processing of the gold-containing arsenopyrite concentrates and may be toxic to microorganisms involved in industrial operations, our research also focused on the arsenic resistance and unique arsenic resistance genes identified in the genome of the LMS strain.

The arsenic resistance (*ars*) operon in microorganisms encodes a transport system that extrudes arsenate, arsenite, and antimonite from the cells, lowering the intracellular concentration of toxic anions. The *ars* operons encoded by plasmids are known to confer plasmid-mediated resistance of *Ac. multivorum* AIU301, *Escherichia (E.) coli*, and *Staphylococcus (St.) aureus* to arsenic and antimony [36]. The arsenic resistance (*ars*) operon from plasmid pKW301 of *Ac. multivorum* AIU 301 conferred resistance of *E. coli* to arsenate and arsenite when overexpressed in *E. coli cells* [36]. Moreover, another arsenic resistance operon (*arsRCB) and the* gene encoding ArsH (arsenical resistance protein) are present in all clades of the genus *Acidiphilium* [9], *including Ac. multivorum LMS* (*contig 3*, ICJ77_02625, ICJ77_02630, and ICJ77_02635). The proteins encoded by the *arsR*, *arsC*, and *arsB* genes of the LMS strain showed the highest amino acid (a. a.) similarity to those of *Ac. multivorum* AIU301 and *Ac. cryptum* JF-5: 99.2, 100, and 99.8% (100% coverage), respectively. The percentage of identity (a. a.) to the corresponding proteins of other *Acidiphilium* spp. strains was 77.9–99.8% (95–100% coverage).

In general, *Acidiphilium* genomes were shown to harbor an abundant repertoire of horizontally transferred genes contributing to environmental adaption, which suggests that the genus *Acidiphilium* originated in mild conditions and adapted to extreme environments after the acquisition of diverse essential functions [9]. In contrast to the *arsRCB operon, which is common for all studied *Acidiphilium* genomes, the* *arsR*, *arsD, arsA*, and *arsB* genes of the probable *arsRDABC* operon of the LMS strain shared similarity with phylogenetically distinct microorganisms and were strain-specific within the genus *Acidiphilium (*Table S1*).* These genes were probably derived by the LMS strain via the horizontal gene transfer (HGT) from other metal-resistant microorganisms. The arsenical resistance operon repressor ArsR and the arsenical resistance operon trans-acting repressor ArsD showed the closest similarity to those of *Pseudaminobacter (Ps.) arsenicus*: 83.2 and 83.9% a. a. similarity, respectively. The arsenic-resistant strain *Ps. arsenicus* CB3^T^ (pH range for growth 6.0–9.0) was isolated from arsenic-rich aquifers of the Jianghan Plain (Hubei, China), the groundwater of which is severely contaminated by arsenic [37]. The arsenical pump-driving ATPase ArsA involved in the removal of arsenate, antimonite, and arsenate from the cell showed the highest similarity to that of *Acetobacteraceae* bacterium SCN 69-10 (85.4% a. a. identity, 100% coverage). Other close homologs of the ArsA pump belonged to *Ps. arsenicus, At. caldus*, *At. ferrivorans*, *At. ferrooxidans*, and *L. ferriphilum* (72.0–85.4% a. a. similarity, 99% coverage). As mentioned above, *Acidithiobacillus* and *Leptospirillum* strains are common members of acidophilic communities in biomining operations, including the processing of gold-bearing pyrite-arsenopyrite concentrates [15,18,19,20,21]. The closest homolog of the arsenical efflux pump membrane protein ArsB was found to belong to the *Gemmataceae* bacterium (94% a. a. similarity, 100% coverage).

Research into the arsenic tolerance of biomining microorganisms has indicated that the arsenic efflux system is an important pathway of arsenite detoxification in *L. ferriphilum* and At. thiooxidans strains [38]. The overall response of *Sb. thermotolerans* cells to the toxic amounts of the gold-containing arsenopyrite concentrate was shown to involve mainly the mechanisms for resistance to arsenic [19]. The acquisition of additional arsenic resistance determinants by At. caldus and *L. ferriphilum* strains may be associated with their improved growth under arsenic stress [35]. In this study, *Ac. multivorum* LMS that was isolated from the pulp of the gold-containing sulfidic concentrate also carried additional arsenic resistance genes, which probably gave this strain an advantage over some other acidophiles in the microbial community and might explain its predominance in the oxidation processes of the pyrite-arsenopyrite concentrates.

Among other proteins specific for the LMS strain within the genus *Acidiphilium*, one protein related to heavy metal resistance was identified (contig 76, ICJ77_17235, Table S1). The closest homolog of this protein belonged to the copper and cadmium translocating P-type ATPase (ZntA) of *Acidocella (Ad.) aminolytica* (89.5% a. a. identity, 99% coverage). The genera *Acidocella* and *Acidiphilium* form two clusters within the alpha-I subclass of the *Proteobacteria* [39]. ZntA couples the hydrolysis of ATP with the export of zinc, cadmium, or lead; it can also bind nickel, copper, cobalt, and mercury [40].

### 3.4. Sulfur Metabolism

**Figure 6:**
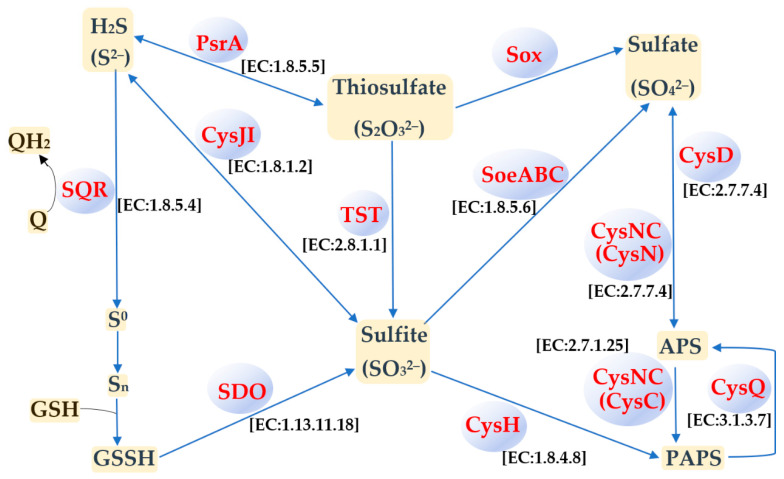
Sulfur metabolism pathways predicted from the genome of *Ac. multivorum* LMS annotated using the NCBI Prokaryotic Genome Annotation Pipeline and KOALA (KEGG Orthology And Links Annotation) tools [31]. SQR: Sulfide:quinone oxidoreductase (EC:1.8.5.4); SDO: Sdo and Sdo1 sulfur dioxygenases (EC:1.13.11.18); Cys JI: CysJ sulfite reductase (NADPH) flavoprotein alpha-component (EC:1.8.1.2) and CysI sulfite reductase (NADPH) hemoprotein beta-component (EC:1.8.1.2); TST: thiosulfate/3-mercaptopyruvate sulfurtransferase (EC:2.8.1.1 2.8.1.2); PsrA: Thiosulfate reductase/polysulfide reductase chain A (EC:1.8.5.5); SoeABC: Sulfite dehydrogenase (quinone) subunit SoeA (EC:1.8.5.6), sulfite dehydrogenase (quinone) subunit SoeB, and sulfite dehydrogenase (quinone) subunit SoeC; Sox: Sox multi-enzyme system that consists of sulfane dehydrogenase subunit SoxC, S-disulfanyl-l-cysteine oxidoreductase SoxD (EC:1.8.2.6), l-cysteine S-thiosulfotransferase SoxX (EC:2.8.5.2), sulfur-oxidizing protein SoxY, sulfur-oxidizing protein SoxZ, l-cysteine S-thiosulfotransferase SoxA (EC:2.8.5.2), and S-sulfosulfanyl-l-cysteine sulfohydrolase SoxB (EC:3.1.6.20); CysNC: Bifunctional enzyme CysN/CysC (EC:2.7.7.4 2.7.1.25); CysD: Sulfate adenylyltransferase subunit 2 (EC:2.7.7.4); CysH: Phosphoadenosine phosphosulfate reductase/phosphoadenylyl-sulfate reductase (EC:1.8.4.8 1.8.4.10); CysQ: 3′(2′),5′-Bisphosphate nucleotidase (EC:3.1.3.7); Q: Quinones; QH_2_: Quinol pool; GSH: Glutathione; GSSH: Glutathione persulfide; S^0^: Elemental sulfur; S_n_: Polysulfide; APS: Adenosine-5′-phosphosulfate; PAPS: Phosphoadenosine phosphosulfate. Substrates, intermediates, and products are indicated in yellow, and proteins catalyzing the reactions are indicated in blue.

Metabolic pathways of *Acidiphilium* strains assigned to different phylogenetic clades were compared in a recent study [9], and different components of the sulfur, nitrogen, and carbon metabolic pathways were shown to be acquired through HGT [9]. In the genome of *Ac. multivorum* LMS, we also determined the genes responsible for dissimilatory and assimilatory nitrate reduction, denitrification, and nitrification. KEGG pathways for carbon metabolism (including methane metabolism) in the LMS strain were also predicted. However, in this study, we focused on the metabolic ability of the LMS strain to oxidize RISCs, which is an unusual feature of the heterotrophic species *Ac. multivorum*. Analysis of the genome of *Ac. multivorum* LMS allowed the determination of the genes encoding different proteins involved in the oxidation of elemental sulfur, sulfite, thiosulfate, and sulfide (Table S2). Based on the NCBI and KEGG annotations, as well as the ability of the LMS strain to oxidize RISCs under mixotrophic and autotrophic conditions, a biochemical model for sulfur oxidation in *Ac. multivorum* LMS was proposed (Figure 6).

Previous analyses of the probable sulfur metabolic pathways predicted from the genome annotations have shown that certain components of sulfur metabolism are encoded by all *Acidiphilium* genomes [9]. In *Ac. multivorum* LMS, the Sox multi-enzyme complex encoded by the *soxCDXYZAB* gene cluster was found (Table S2). As in the case of other *Acidiphilium* strains assigned to clade IV [9], including the closest phylogenetic relative *Acidiphilium* sp. AccII, the Sox multi-enzyme complex of *Ac. multivorum* LMS was likely derived from the most recent common ancestor of clades III and IV. This conclusion was supported by the previous data [9], as well as the highest similarity of the Sox system components of the LMS strain (Table S2) to those of the members of the genus *Acidiphilium* (up to 98–100% a. a. identity, 100% coverage). In contrast to the Sox complexes of clades III and IV, those of clades I and II were probably acquired via independent HGT events [9]. The typical periplasmic multi-enzyme Sox system is involved in the oxidation of thiosulfate, sulfide, sulfite, and elemental sulfur to produce sulfate as the final product [41]. In *Acidithiobacillus* strains (the most well-studied sulfur- and iron-oxidizing acidophiles), the Sox-pathway-dependent sulfur oxidation is known to provide higher sulfur oxidation capacities (higher S^0^-oxidizing rate and biomass) compared to Sox-deficient strains [41].

Three genes coding for thiosulfate/3-mercaptopyruvate sulfurtransferase (TST) (EC:2.8.1.1 2.8.1.2) (ICJ77_05465 (contig 7), ICJ77_08765 (contig 13), and ICJ77_09760 (contig 16); Table S2) were also identified in the genome of the LMS strain. Rhodanase (TST) catalyzes the pathway of S_2_O_3_^2−^ utilization, producing sulfite [41] (Figure 6).

Among other enzymes involved in sulfur oxidation, sulfur dioxygenases (SDOs) were predicted from the annotated genome (Table S2; Figure 6). Possibly, SDOs play an essential role in sulfur oxidation by *Ac. multivorum* LMS. The oxidation of S^0^ to sulfite, catalyzed by SDO, was first reported in *At. ferrooxidans* [42]. Further studies showed that SDO used the sulfane sulfur atom of glutathione persulfide (GSSH) (and not S^0^) as a substrate to produce sulfite [43]. Although SDO activity was first reported in 1987 [42] and the actual substrate of SDO was determined in 2003 [43], the *sdo* genes in *Acidithiobacillus* spp. were identified only several years ago [44]. Moreover, a new subgroup of SDOs was recently identified in *At. caldus*. The previously identified SDO group [44] and the new one were designated Sdo2 and Sdo1, respectively [45].

As in the case of *Acidiphilium* spp. strains (clades I, III, and IV) [9], a homolog of the new Sdo1 associated with tetrathionate oxidation in *At. caldus* MTH-04 (Sdo1, A5904_0421) [45] was identified in the genome of *Ac. multivorum* LMS (Table S2). A comparison using the NCBI BLAST revealed a 49.4% a. a. identity (100% coverage) between Sdo1 of *At. caldus* MTH-04 and a metallo-beta-lactamase family protein of the LMS strain (ICJ77_14405, contig 38; Table S2). Moreover, we also identified a homolog of Sdo2 of *At. caldus* MTH-04 (A5904_0790, [45]) in the LMS strain (ICJ77_00340, contig 1; Table S2), with a 35% a. a. similarity (93% coverage) between them. Interestingly, a strong correlation between the *sdo1* gene coding for Sdo1 and the tetrathionate intermediate pathway was previously revealed in *At. caldus* MTH-04 [45]. The deletion of *sdo2* promoted bacterial growth on tetrathionate and thiosulfate, while the overexpression of *sdo2* altered gene expression patterns of SQR and TST. Thus, *sdo1* is essential for the survival of *At*. *caldus* when tetrathionate is used as the sole energy source, and *sdo2* may also play a role in sulfur metabolism [45]. BLAST searching carried out in this study revealed homologs of Sdo2 of the LMS strain also in other *Acidiphilium* strains: *Ac. cryptum* JF-5, *Acidiphilium* sp. PM, and *Acidiphilium* sp. JA12-A1 (100% a. a. identity, 100% coverage); *Ac. multivorum* AIU301^T^ (99.3% a. a. identity, 100% coverage); *Acidiphilium* spp. C61 and 37-67-22 (90.7–91.0% a. a. identity, 100% coverage); as well as *Ac. rubrum* (82% a. a. identity, 100% coverage) and *Acidiphilium* sp. 37-64-53 (81.7% a. a. identity, 100% coverage). According to the previously proposed phylogenetic classification by Li et al. [9], these strains belong to clades II–IV [9].

Thus, we propose an SDO-dependent and Sox-pathway-dependent sulfur oxidation model functioning in the LMS strain (Figure 6). According to this model, extracellular S^0^ is first activated by thiol-containing outer-membrane proteins to generate persulfide sulfane sulfur, and the latter is further oxidized by periplasmic SDO to produce sulfite as previously described [43]. Sulfite is oxidized in the APS (adenosine-5′-phosphosulfate)-mediated process, with the participation of Cys proteins, and by the heterotrimeric membrane-bound complex SoeABC (Figure 6). Genes encoding homologs of sulfite reductase subunit beta (CysI) and sulfate adenylyltransferase (CysD) of the LMS strain have been previously found to be upregulated in *Acidiphilium cryptum* DX1-1 that was grown under mixotrophic conditions in the culture medium containing glucose and sulfur [6].

## 4. Conclusions

In the present research, we isolated and characterized the LMS strain involved in the large-scale industrial processing of the gold-bearing pyrite-arsenopyrite ore concentrate at the Olimpiada biooxidation plant (Siberia, Russia). Surprisingly, the LMS strain that was assigned to the heterotrophic species *Ac. multivorum* proved to be among predominant sulfur oxidizers in the commercial acidophilic microbial communities in bioreactors. Summarizing data of several years of monitoring the industrial microbial populations, we concluded that *Ac. multivorum* was able to become a predominant sulfur oxidizer together with more common sulfur-oxidizing acidophiles, such as *At. thiooxidans*, or even succeed the latter in the commercial microbial communities. In this study, it was shown that:

- *Ac. multivorum* LMS proved to be one of the predominant sulfur oxidizers in the microbial populations during industrial processing of the gold-bearing pyrite-arsenopyrite ore concentrates;
- Phenotypic features of LMS strain included wide pH and temperature ranges for growth, as well as the ability to grow organotrophically, lithotrophically, or mixotrophically;
- The first draft genome of the sulfur-oxidizing *Ac. multivorum* was sequenced, annotated, and compared to other *Acidiphilium* genomes available in the databases;
- Based on the functional analysis of the genome and sulfur-oxidizing activity of the strain, a biochemical model for sulfur oxidation in *Ac. multivorum* LMS was proposed;
- Strain-specific genes of *Ac. multivorum* LMS may confer additional resistance to arsenic and heavy metals, such as copper, cadmium, and zinc;
- The commercial importance of *Ac. multivorum* LMS was proved by the efficient sulfur oxidation in the presence or absence of organic compounds, a complicated system of sulfur oxidation pathways, as well as additional metal resistance determinants specific for the LMS strain within the genus *Acidiphilium*.

Overall, the phenotypic features of *Ac. multivorum* LMS determined in this study, as well as functional analysis of its genome, made it possible to conclude that this microorganism plays a previously underestimated central role in acidophilic communities during biooxidation of gold-bearing sulfidic raw materials. Phenotypic characteristics, such as wide pH and temperature ranges for growth, the ability to oxidize RISCs, as well as versatile metabolic and resistance capacities, gave the LMS strain a competitive advantage in microbial communities of industrial stirred tank bioreactors and determined its predominance in commercial microbial populations.

## Figures and Tables

**Table 1:** Main phenotypic features of *Ac. multivorum* LMS compared to characteristics of the type strain *Ac. multivorum* AIU301^T^.

Parameter	LMS ^1^	AIU301 ^2^
Source of isolation	Industrial bioreactor	AMD ^3^
Morphology	Rods, single cells, and aggregates	Rods, single cells, and chains
Cell size, µm	0.3–0.5 × 0.8–1.0	0.5–1.2 × 0.9–3.8
Motility	+	+
pH range	1.6–5.5	1.9–5.6
pH_opt_^4^	2.5–4.3	3.2–4.0
*T*^5^ range, °C	<20–47	17–42
*T*_opt_, °C	30–40	27–35
*µ_m_*_ax_^6^_,_ h^−1^	0.02 (S^0^ + YE^7^)	0.004–0.062 (organic substrates)
Growth with:		
YE ^7^	+	+
Glucose	+	+
YE + S^0^	+	–
S^0^	+	–
YE + Na_2_S_2_O_3_	+	–
Na_2_S_2_O_3_	+	–
Fe^2+^	–	–
Reference	(present study)	[2]

^1^ LMS, *Ac. multivorum* LMS (present study); ^2^ AIU301, *Ac. multivorum* AIU301^T^; ^3^ AMD, acid mine drainage; ^4^ opt, optimum; ^5^ *T*, temperature; ^6^ *µ_m_*_ax_, maximum specific growth rate; ^7^ YE, yeast extract.

**Table 2:** Statistics of the genome of *Ac. multivorum* LMS.

Attribute	Value
Genome size (bp)	3,837,559
Number of contigs	139
Number of contigs (≥1000 bp)	135
Number of contigs (≥5000 bp)	84
Number of contigs (≥10,000 bp)	62
Number of contigs (≥25,000 bp)	43
Number of contigs (≥50,000 bp)	23
Largest contig	308,818
DNA, G + C (%)	67.3
Genes (total)	3676
CDSs (total)	3623
Genes (coding)	3492
CDSs (with protein)	3492
Genes (RNA)	53
Complete rRNAs	1, 1, 1 (5S, 16S, 23S)
tRNAs	46
ncRNAs	4
Pseudogenes (total)	131
CRISPR arrays	1

**Table 3:** Results of the ortholog annotation of the proteins encoded by the genome of *Ac. multivorum* LMS (56.1% annotated) using KOALA (KEGG Orthology And Links Annotation) tools [31].

Functional Category	Number of Proteins ^1^
Carbohydrate metabolism	242
Protein families: Genetic information processing	235
Environmental information processing	226
Protein families: Signaling and cellular processes	213
Genetic information processing	164
Amino acid metabolism	157
Unclassified: Metabolism	134
Energy metabolism	117
Metabolism of cofactors and vitamins	104
Unclassified	103
Cellular processes	100
Nucleotide metabolism	63
Unclassified: Genetic information processing	55
Protein families: Metabolism	53
Lipid metabolism	46
Glycan biosynthesis and metabolism	39
Unclassified: Signaling and cellular processes	39
Xenobiotics biodegradation and metabolism	36
Metabolism of other amino acids	28
Metabolism of terpenoids and polyketides	14

^1^ Number of proteins encoded by *Ac. multivorum* LMS that are associated with the main functional categories.

**Table 4:** The average nucleotide identity (ANI) between the genomes of *Ac. multivorum* LMS and the most closely related *Acidiphilium* strains. The ANI values were calculated with the ANI calculator (Kostas lab, Atlanta, GA, USA) using reciprocal best hits (two-way ANI).

*Acidiphilium* Strain	ANI Value (SD ^1^), %
*Acidiphilium* sp. AccII	99.29 (1.43)
*Acidiphilium* sp. JA12-A1	99.23 (0.83)
*Ac. multivorum* AIU301^T^	99.12 (1.92)
*Ac. cryptum* JF-5	99.18 (1.02)
*Acidiphilium* sp. ZJSH63	99.04 (1.52)
*Acidiphilium* sp. AccI	98.97 (1.84)

^1^ SD, standard deviation.

## Data Availability

The genome sequence of *Ac. multivorum* LMS was deposited in the NCBI databases under the accession number JACVVW000000000 (BioProject PRJNA662208; BioSample SAMN16078398). The 16S rRNA gene sequences of the *Acidiphilium* sp. strains PB-B3-4, PB-B1-3, PB-B2-3, and LMS were deposited in the GenBank databases under accession numbers MW389315–MW389318.

## Supplementary Materials

The following are available online at https://www.mdpi.com/article/10.3390/microorganisms9050984/s1, Table S1: Unique genes and corresponding proteins, identified in the genome of *Ac. multivorum* LMS. A comparison to other *Acidiphilum* genomes was carried out by analysis of core genome, pangenome, and accessory genomic regions, Table S2: Sulfur metabolism components predicted from the genome of *Ac. multivorum* LMS. Ortholog annotation was carried out using KOALA (KEGG Orthology And Links Annotation) system. Genome annotation was performed using the NCBI Prokaryotic Genome Annotation Pipeline and RAST (Rapid Annotation using Subsystem Technology service version 2.0 RASTtk annotation scheme).
