# Supplementary material for: Unraveling the Central Role of Sulfur-Oxidizing Acidiphilium multivorum LMS in Industrial Bioprocessing of Gold-Bearing Sulfide Concentrates
Source: Microorganisms. 2021 May 1;9(5):984. doi: 10.3390/microorganisms9050984 (PMC8147356; doi:10.3390/microorganisms9050984)
Supplement: Supplementary file 1 [file microorganisms-09-00984-s001.zip › microorganisms-1164548-supplementary.docx]

**Supplementary Materials**

Anna Panyushkina, Aleksandr Bulaev and Aleksandr V. Belyi

**Unraveling the Central Role of Sulfur-Oxidizing *Acidiphilium multivorum* LMS in Industrial Bioprocessing of Gold-Bearing Sulfide Concentrates**

**Table S1.** Unique genes and corresponding proteins, identified in the genome of *Ac. multivorum* LMS. A comparison to other *Acidiphilum* genomes was carried out by analysis of core genome, pangenome, and accessory genomic regions.

| No. | Contig No.  (Node, NCBI Annotation) | Locus Position  (NCBI Annotation) | CDS (Protein ID) | Product (NCBI Annotation) | Homolog(s), Closest Organism(s); Percentage Identity (Query Coverage) | Description; Putative Function(s) |
| --- | --- | --- | --- | --- | --- | --- |
| 1 | 23 | 8975–14290 | ICJ77_11685 | Hypothetical protein | DUF 3883 domain-containing protein, *Mesorhizobium* sp.; 57.9% (98%) | Uncharacterized protein containing DUF 3883 domain of unknown function; it is found on restriction endonucleases. |
| 2 | 23 | 38535–41732 | ICJ77_11820 | Helicase | Helicase, сandidatus *Nitrospira nitrificans*; 79,3% (99%) | Superfamily II DNA or RNA helicase, SNF2 family. Transcription, replication, recombination, and repair. |
| 3 | 23 | 42499–44469 | ICJ77_11830 | DUF262 domain-containing protein | DUF262 domain-containing protein, *Methylocystis parvus*; 80.6% (100%) | Uncharacterized conserved protein containing ParB-like and HNH nuclease domains. Function unknown. |
| 4 | 23 | 44516–46426 | ICJ77_11835 | Site-specific DNA-methyl-transferase | Site-specific DNA-methyltransferase, *Rhodocyclaceae* bacterium; 72% (100%) | Adenine specific DNA methylase Mod (Replication, recombination, and repair) S-adenosylmethionine-dependent methyltransferases, class I, use S-adenosyl-L-methionine as a substrate for methyltransfer, creating the product S-adenosyl-L-homocysteine. |
| 5 | 23 | 48144–51138 | ICJ77_11850 | DEAD/DEAH box helicase family protein | DEAD/DEAH box helicase family protein, *Methylococcus* sp. IM1; 78.3% (98%) | Type III restriction-modification system endonuclease. Defense mechanisms. |
| 6 | 39 | 27978–30884 | ICJ77_14645 | Hypothetical protein | Hypothetical protein, *Paracoccus versutus*; 58.7% (99%) | Unknown function. |
| 7 | 80 | 70–387 | ICJ77_17350 | Cation transporter | Cation transporter, *Rhodospirillales* bacterium 69-11; 61.9% (91%) | Co/Zn/Cd efflux system component, transports divalent cations of cobalt, cadmium and/or zinc |
| 8 | 42 | 4516–7719 | ICJ77_14930 | Hypothetical protein | Hypothetical protein EOR67_21590, *Mesorhizobium* sp.; 85.1% (98%) | KAP family P-loop domain; The KAP family of predicted NTPases are sporadically distributed across a wide phylogenetic range in bacteria and in animals. Many of the prokaryotic KAP NTPases are encoded in plasmids and tend to undergo disruption to form pseudogenes. |
| *9* | 42 | 8172–10151 | ICJ77_14935 | Conjugal transfer protein TraG | Conjugal transfer protein, *Rhodopila globiformis*; 89.2% (100%) | Type IV secretory pathway, VirD4 component, TraG/TraD family ATPase. Type IV secretory system; conjugative DNA transfer. These proteins contain a P-loop and walker-B site for nucleotide binding. TraG is essential for DNA transfer in bacterial conjugation. These proteins are thought to mediate interactions between the DNA-processing (Dtr) and the mating pair formation (Mpf) systems. The C-terminus of this domain interacts with the relaxosome component TraM via the latter's tetramerisation domain. TraD is a hexameric ring ATPase that forms the cytoplasmic face of the conjugative pore. The family contains a number of different DNA transfer proteins. |
| 10 | 42 | 12382..14820 | ICJ77_14960 | Conjugal transfer protein TrbE | Conjugal transfer protein TrbE, *Novosphingobium naphthalenivorans*; 92% (99%) | Conjugal transfer ATPase TrbE; Provisional. Type IV secretion/conjugal transfer ATPase, VirB4 family. Type IV secretion systems are found in Gram-negative pathogens. They export proteins, DNA, or complexes in different systems and are related to plasmid conjugation systems. This model represents related ATPases that include VirB4 in Agrobacterium tumefaciens (DNA export) CagE in Helicobacter pylori (protein export) and plasmid TraB (conjugation). |
| 11 | 42 | 22354–24060 | ICJ77_15010 | Carboxylesterase family protein | Carboxylesterase family protein, *Methylocella tundrae*; 56.2% (98%) | Esterases and lipases that act on carboxylic esters. |
| 12 | 4 | 7288–8715 | ICJ77_03325 | Hypothetical protein | Hypothetical protein B7Z75_08180, *Acidocella* sp. 20-57-95; 44.4% (98%) | Function unknown. |
| 13 | 51 | 5282–9181 | ICJ77_15890 | Relaxase domain-containing protein | Relaxase domain-containing protein, *Acetobacteraceae bacterium*; 57.5% (85%) | Conjugative transfer relaxase protein TraI. This protein is a component of the relaxosome complex. In the process of conjugative plasmid transfer, the realaxosome binds to the plasmid at the oriT (origin of transfer) site. The relaxase protein TraI mediates the single-strand nicking and ATP-dependent unwinding (relaxation, helicase activity) of the plasmid molecule. These two activities reside in separate domains of the protein. |
| 14 | 53 | 3086–5260 | ICJ77_16010 | Sigma-70 family RNA polymerase sigma factor | RNA polymerase subunit sigma-70, *Tabrizicola* sp. TH137; 75.4 (100%) | Transcription, Transcription factors. |
|  |  |  |  |  |  |  |
| 15 | 53 | 5260–6762 | ICJ77_16015 | ATP-binding protein | Endonuclease, *Rhizobiales bacterium*; 94.2% (100%) | Histidine kinase-, DNA gyrase B-, and HSP90-like ATPase. This family represents, additionally, the structurally related ATPase domains of histidine kinase, DNA gyrase B and HSP90. |
| 16 | 53 | 9636–10640 | ICJ77_16025 | PD-(D/E)XK motif protein | Hypothetical protein, *Salinicola* sp.; 87.7% (100%) | Putative PD-(D/E)XK family member, (DUF4420) ;This family of proteins is functionally uncharacterized. |
| 17 | 53 | 10642–12447 | ICJ77_16030 | AIPR family protein | AIPR protein, *Rhodovulum* sp. NI22; 94.7% (99%) | This family of proteins was identified in as an abortive infection phage resistance protein often found in restriction modification system operons. |
| 18 | 53 | 12510–14045 | ICJ77_16035 | DNA cytosine methyltransferase | DNA cytosine methyltransferase, *Amorphus coralli*; 82.9% (99%) | Site-specific DNA-cytosine methylase [Replication, recombination and repair]; Cytosine-C5 specific DNA methylases; Methyl transfer reactions play an important role in many aspects of biology. Cytosine-specific DNA methylases are found both in prokaryotes and eukaryotes. |
| 19 | 53 | 14113–14598 | ICJ77_16040 | DNA mismatch endonuclease Vsr | DNA mismatch endonuclease Vsr, *Rhodovulum* sp. NI22; 82.1% (93%) | Very Short Patch Repair (Vsr) Endonuclease. Endonucleases in DNA repair that recognize damaged DNA and cleave the phosphodiester backbone. Vsr endonucleases have a common endonuclease topology that has been tailored for recognition of TG mismatches. |
|  |  |  |  |  |  |  |
| 20 | 58 | 4253–6175 | ICJ77_16365 | HAMP domain-containing protein | Methyl-accepting chemotaxis protein, one *Acidiphilium* sp. strain 37-64-53); 60.2% (91%); *Acidocella aminolytica*, 57.8% (90%) | Methyl-accepting chemotaxis protein [Cell motility, Signal transduction mechanisms]. Methyl-accepting chemotaxis-like domains (chemotaxis sensory transducer); Thought to undergo reversible methylation in response to attractants or repellants during bacterial chemotaxis. |
| 21 | 68 | 24–2129 | ICJ77_16920 | AAA family ATPase | Hypothetical protein B7Z75_13330, *Acidocella* sp. 2057-95 | Ti-type conjugative transfer relaxase TraA. This protein contains domains distinctive of a single strand exonuclease (N-terminus, MobA/MobL, pfam03389) as well as a helicase domain (central region, homologous to the corresponding region of the F-type relaxase TraI, TIGR02760). This protein likely fills the same role as TraI(F), nicking (at the oriT site) and unwinding the coiled plasmid prior to conjugative transfer. |
| 22 | 68 | 2465–2860 | ICJ77_16925 | Hypothetical protein | Conjugal transfer protein TraD, *Acidocella aromatica*; 40.2% (66%) | Conjugal transfer. |
| 23 | 68 | 3138–3764 | ICJ77_16930 | EI24 domain-containing protein | EI24 domain-containing protein, *Acidisphaera* sp. S103; 60.2% (98%) | Etoposide-induced protein 2.4 (EI24). This family contains a number of eukaryotic etoposide-induced 2.4 (EI24) proteins approximately 350 residues long as well as bacterial CysZ proteins (formerly known as DUF540). In cells treated with the cytotoxic drug etoposide, EI24 is induced by p53. It has been suggested to play an important role in negative cell growth control. PRK12768, sulfate transporter family protein. |
| 24 | 86 | 2594–4582 | ICJ77_17545 | ParB N-terminal domain-containing protein | ParB N-terminal domain-containing protein *Acidibrevibacterium fodinaquatile*; 90.3% (99%); one *Acidiphilium* sp. strain C61; 67.2% (98%) | ParB N-terminal, parA-binding, -like domain of bacterial and plasmid parABS partitioning systems ;This family represents the N-terminal domain of ParB, a DNA-binding component of the prokaryotic parABS partitioning system. parABS contributes to the efficient segregation of chromosomes and low-copy number plasmids to daughter cells during prokaryotic cell division. |
| 25 | 98 | 32–430 | ICJ77_17765 | Winged helix-turn-helix transcriptional regulator | Winged helix-turn-helix transcriptional regulator, *Pseudaminobacter arsenicus*; 83.2% (76%) | Arsenical Resistance Operon Repressor and similar prokaryotic metal-regulated homodimeric repressors. ARSR subfamily of helix-turn-helix bacterial transcription regulatory proteins (winged helix topology). Includes several proteins that appear to dissociate from DNA in the presence of metal ions. |
| 26 | 98 | 516–887 | ICJ77_17770 | ArsD, arsenite efflux transporter metallochaperone | Arsenite efflux transporter metallochaperone ArsD *Pseudaminobacter arsenicus*; 83.9% (100%) | Arsenical resistance operon trans-acting repressor ArsD. ArsD is a trans-acting repressor of the arsRDABC operon that confers resistance to arsenicals and antimonials. It possesses two pairs of vicinal cysteine residues, Cys(12)-Cys(13) and Cys(112)-Cys(113), that potentially form separate binding sites for the metalloids that trigger dissociation of ArsD from the operon. However, as a homodimer it has four vicinal cysteine pairs. |
| 27 | 98 | 898–2652 | ICJ77_17775 | ArsA, arsenical pump-driving ATPase | Arsenical pump-driving ATPase, *Acetobacteraceae* bacterium SCN 69-10; 85.4% (100%), *Acidithiobacillus caldus*; 79.4% (99%) | Pfam02374 anion-transporting ATPase involved in the removal of arsenate, antimonite, and arsenate from the cell. |
| 28 | 98 | 3138–3783 | ICJ77_17785 | ArsB, arsenical efflux pump membrane protein | TPA: arsenical efflux pump membrane protein ArsB, *Gemmataceae* bacterium; 94% (100%) | Arsenite/antimonite efflux pump membrane protein. Members of this protein family are ArsB, a highly hydrophobic integral membrane protein involved in transport processes used to protect cells from arsenite (or antimonite). |
| 29 | 68 | 4055–4642 | ICJ77_16935 | Hypothetical protein | DotA/TraY family protein, *Azospirillum* sp. TSA6c; 45.5% (68%) | Conjugal transfer/type IV secretion protein DotA/TraY. Members of this protein family include transfer protein TraY of IncI1 plasmid R64 and DotA (defect in organelle trafficking A) of Legionella pneumophila. |
| 30 | 76 | 1489–3924 | ICJ77_17235 | Cation-translocating P-type ATPase | ZntA, cation-translocating P-type ATPase, *Acidocella aminolytica*; 89.5% (99%) | Heavy metal translocating P-type ATPase. This model encompasses two equivalog models for the copper and cadmium-type heavy metal transporting P-type ATPases (TIGR01511 and TIGR01512) as well as those species which score ambiguously between both models. |
| 31 | 7 | 120826–121023 | ICJ77_06080 | AlpA family transcriptional regulator | AlpA family transcriptional regulator, the only one *Acidiphilium* sp. strain PM; 87.1% (95%); AlpA family transcriptional regulator, *Ensifer aridi*; 55.9% (90%) | Predicted DNA-binding transcriptional regulator AlpA. Transcription, Mobilome: prophages, transposons. |
| 32 | 7 | 121594–124077 | ICJ77_06085 | DUF3987 domain-containing protein | Hypothetical protein B7Z75_13270, *Acidocella* sp. 20-57-95; 57.4% (57%); DUF3987 domain-containing protein, the only one *Acidiphilium* sp. strain C61 41.3% (70%) | Uncharacterized protein, contains primase-polymerase (Primpol) domain. Function unknown. |

**Table S2.** Sulfur metabolism components predicted from the genome of *Ac. multivorum* LMS. Ortholog annotation was carried out using KOALA (KEGG Orthology And Links Annotation) system. Genome annotation was performed using the NCBI Prokaryotic Genome Annotation Pipeline and RAST (Rapid Annotation using Subsystem Technology service version 2.0 RASTtk annotation scheme).

| CDS (Protein ID); Contig No. | KO ^1^ | Protein size, a. a. | Putative homolog(s) | Score |
| --- | --- | --- | --- | --- |
| ICJ77_01505; 2 | K00955 | 621 | *cysNC*; bifunctional enzyme CysN/CysC [EC:2.7.7.4 2.7.1.25] | 414 |
| ICJ77_01500; 2 | K00957 | 308 | *cysD*; sulfate adenylyltransferase subunit 2 [EC:2.7.7.4] | 293 |
| ICJ77_01860; 2 | K17218 | 435 | *sqr*; sulfide:quinone oxidoreductase [EC:1.8.5.4] | 294 |
| ICJ77_02570; 3 | K00380 | 582 | *cysJ*; sulfite reductase (NADPH) flavoprotein alpha-component [EC:1.8.1.2] | 271 |
| ICJ77_02565; 3 | K00390 | 227 | *cysH*; phosphoadenosine phosphosulfate reductase/ phosphoadenylyl-sulfate reductase [EC:1.8.4.8 1.8.4.10] | 142 |
| ICJ77_02560; 3 | K00381 | 557 | *cysI*; sulfite reductase (NADPH) hemoprotein beta-component [EC:1.8.1.2] | 396 |
| ICJ77_05475; 7 | K08352 | 731 | *phsA*, *psrA*; thiosulfate reductase / polysulfide reductase chain A [EC:1.8.5.5] | 393 |
| ICJ77_05465; 7 | K01011 | 333 | TST, MPST, *sseA*; thiosulfate/3-mercaptopyruvate sulfurtransferase [EC:2.8.1.1 2.8.1.2] | 154 |
| ICJ77_08765; 13 | K01011 | 313 | TST, MPST, *sseA*; thiosulfate/3-mercaptopyruvate sulfurtransferase [EC:2.8.1.1 2.8.1.2] | 192 |
| ICJ77_09760; 16 | K01011 | 284 | TST, MPST, *sseA*; thiosulfate/3-mercaptopyruvate sulfurtransferase [EC:2.8.1.1 2.8.1.2] | 154 |
| ICJ77_16555 (NCBI annotation, pseudo gene; annotated using RAST and KOALA); 61 | K17225 | 349 | *soxC*; sulfane dehydrogenase subunit SoxC | 185 |
| ICJ77_16560; 61 | K22622 | 259 | *soxD*; S-disulfanyl-L-cysteine oxidoreductase SoxD [EC:1.8.2.6] | 61 |
| ICJ77_16565; 61 | K17223 | 114 | *soxX*; L-cysteine S-thiosulfotransferase [EC:2.8.5.2] | 35 |
| ICJ77_16570; 61 | K17226 | 164 | *soxY*; sulfur-oxidizing protein SoxY | 69 |
| ICJ77_16575; 61 | K17227 | 103 | *soxZ*; sulfur-oxidizing protein SoxZ | 70 |
| ICJ77_16580; 61 | K17222 | 296 | *soxA*; L-cysteine S-thiosulfotransferase [EC:2.8.5.2] | 132 |
| ICJ77_16590; 61 | K17224 | 578 | *soxB*; S-sulfosulfanyl-L-cysteine sulfohydrolase [EC:3.1.6.20] | 366 |
| ICJ77_10675; 19 | K21307 | 935 | *soeA*; sulfite dehydrogenase (quinone) subunit SoeA [EC:1.8.5.6] | 583 |
| ICJ77_10680; 19 | K21308 | 247 | *soeB*; sulfite dehydrogenase (quinone) subunit SoeB | 173 |
| ICJ77_10685; 19 | K21309 | 312 | *soeC*; sulfite dehydrogenase (quinone) subunit SoeC | 153 |
| ICJ77_15445; 46 | K01082 | 256 | *cysQ*, MET22, BPNT1; 3'(2'), 5'-bisphosphate nucleotidase [EC:3.1.3.7] | 135 |
| ICJ77_00340; 1 | – ^2^ | 289 | *sdo2*, MBL fold metallo-hydrolase (SDO, sulfur dioxygenase [EC:1.13.11.18]) | – |
| ICJ77_14405; 38 | – | 243 | *sdo1*, MBL fold metallo-hydrolase (SDO1, sulfur dioxygenase [EC:1.13.11.18]) | – |

^1^ KO, KEGG Orthology identifiers; ^2^ –, not determined.
